# Optimization of central carbon metabolism by Warburg effect of human cancer cell improves triterpenes biosynthesis in yeast

**DOI:** 10.1007/s44307-023-00004-6

**Published:** 2023-10-26

**Authors:** Xiaona Lin, Tianyue An, Danni Fu, Sujuan Duan, Hong-Lei Jin, Hong-Bin Wang

**Affiliations:** 1 Institute of Medical Plant Physiology and Ecology, School of Pharmaceutical Sciences, Guangzhou University of Chinese Medicine, Guangzhou, 510006 China; 2 School of Integrated Traditional Chinese and Western Medicine, Binzhou Medical University, Yantai, 264003 China; 3 Guangzhou Key Laboratory of Chinese Medicine Research On Prevention and Treatment of Osteoporosis, The Third Affiliated Hospital of Guangzhou University of Chinese Medicine, Guangzhou, 510006 China; 4 Key Laboratory of Chinese Medicinal Resource From Lingnan, (Guangzhou University of Chinese Medicine), Ministry of Education, Guangzhou, 510006 China; 5 State Key Laboratory of Dampness Syndrome of Chinese Medicine, Guangzhou University of Chinese Medicine, Guangzhou, 510006 China

**Keywords:** *Saccharomyces cerevisiae*, Metabolic engineering, Central carbon metabolism, Warburg effect, Triterpenes

## Abstract

Optimizing central carbon metabolism (CCM) represents an attractive and challenging strategy to improve the biosynthesis of valuable chemicals due to the complex regulation of the CCM in yeast. In this study, we triggered the similar Warburg effect of cancer cells in yeast strains by introducing the human hypoxia-inducible factor-1 (HIF-1) complex, which regulated the expression of numerous enzymes involved in CCM and redirected the metabolic flux from glycolysis to tricarboxylic acid cycle. This redirection promoted the production of squalene to a 2.7-fold increase than the control strain BY4741. Furthermore, the HIF-1 complex boosted the production of represented endogenous triterpenoid ergosterol to 1145.95 mg/L, and exogenous triterpenoid lupeol to 236.35 mg/L in shake flask cultivation, 10.5-fold and 9.2-fold increase than engineered strains without HIF-1 complex integration, respectively. This study provides a novel strategy for optimizing CCM by HIF-1 mediated Warburg effect of cancer cells to improve biosynthesis of triterpenoids in yeast.

**Supplementary Information:**

The online version contains supplementary material available at 10.1007/s44307-023-00004-6.

## Introduction

Natural products account for more than half of all small-molecule pharmaceutical agents in current clinical use (Patridge et al. 2016). Among them, triterpenes belong to the largest category of plant natural products, and more than 20,000 triterpenes have been discovered (Thimmappa et al. 2014). The subclass of triterpenes can be divided into several structural families according to their basic skeletons, the derivatives of protostane, cycloartane, dammarane, euphane, and pentacyclic derivatives (Bachořík and Urban 2021). Many triterpenes, such as ginsenosides and lupeol, are of particular interest and value. Ginsenosides are known to possess a lot of biological activities, including regulatory effects on immunomodulation, protection functions in the central nervous and cardiovascular systems, anti-diabetic, anti-aging, and anti-carcinogenic (Piao et al. 2020). Lupeol has anti-inflammatory, antioxidant, anticancer, and other pharmacological effects (Badshah et al. 2016; Guo et al. 2018; Min et al. 2019). The derivatives of lupeol, such as betulinic acid, also exert protection of the heart, liver, and skin in some specific disease models (Beserra et al. 2018; Huang et al. 2021; Xu et al. 2020).

Valuable triterpenes can be obtained from processed plant biomass, but it requires substantial land, water, and time investment, and inevitable insecurity in supply chains due to variability in crop yields resulting from pests or extreme weather (Cravens et al. 2019). In the meantime, the complex structure of triterpenes makes them challenging to be chemically synthesized (Thimmappa et al. 2014). Thus, setting up cell factories by using engineered microorganisms is eco-friendly and efficient, and has already provided sustainable and reliable supplies for many natural compounds, including artemisinic acid and taxadiene (Kumaran Ajikumar et al. 2010; Paddon et al. 2013). *Saccharomyces cerevisiae*, a well-studied yeast strain, is equipped with a broad spectrum of molecular engineering tools and is suitable for large-scale genetic operations (Cataldo et al. 2020). The high-level production of artemisinic acid, patchoulol, and ginsenoside in yeast showed the high potential of yeast as a terpenoid cell factory (Ma et al. 2019; Paddon et al. 2013; Wang et al. 2019, 2015). Triterpenes are synthesized from the universal precursor squalene, which is also a widely used compound in pharmaceutical and personal care (Spanova and Daum 2011). In yeast, squalene is produced by the mevalonate (MVA) and sterol biosynthesis pathways using acetyl-CoA as the starting precursor. Since the yeast cytosolic acetyl-CoA can be derived from pyruvate via the pyruvate decarboxylation pathway (Eram and Ma 2013), increasing the accumulation of pyruvate in the cytoplasm could be an effective method to promote the production of squalene.

Notably, the Warburg effect, known as aerobic glycolysis, is a remarkable form of cellular metabolism in cancer cells, with the characteristic of high levels of glucose uptake via the enhanced glycolytic pathway and increased conversion of glucose to lactose even in the presence of oxygen (Koppenol et al. 2011). Besides the regulation of the Warburg effect on the central carbon metabolism (CCM), this effect also prevents the access of pyruvate into mitochondria for oxidative phosphorylation and promotes the degradation of pyruvate into lactate in the cytoplasm (Heiden et al. 2009). Hypoxia-inducible factor-1 (HIF-1) complex, a kind of transcription factor composed of HIF-1α and ARNT (HIF-1β), can mediate and induce the Warburg effect in many types of cancer cells (Wu et al. 2021). Previous reports showed that the human HIF-1 complex could be expressed in yeast cells and exerted similar transcriptional activity in this heterologous host (Braliou et al. 2006). Therefore, we suppose that HIF-1 can increase the accumulation of pyruvate in yeast cytoplasm, and as no lactate dehydrogenase in yeast cells, the enriched pyruvate in the cytoplasm would facilitate the pyruvate dehydrogenase bypass to increase the biosynthesis of acetyl-CoA, and ultimately improve the production of squalene downstream.

**Fig. 1:**
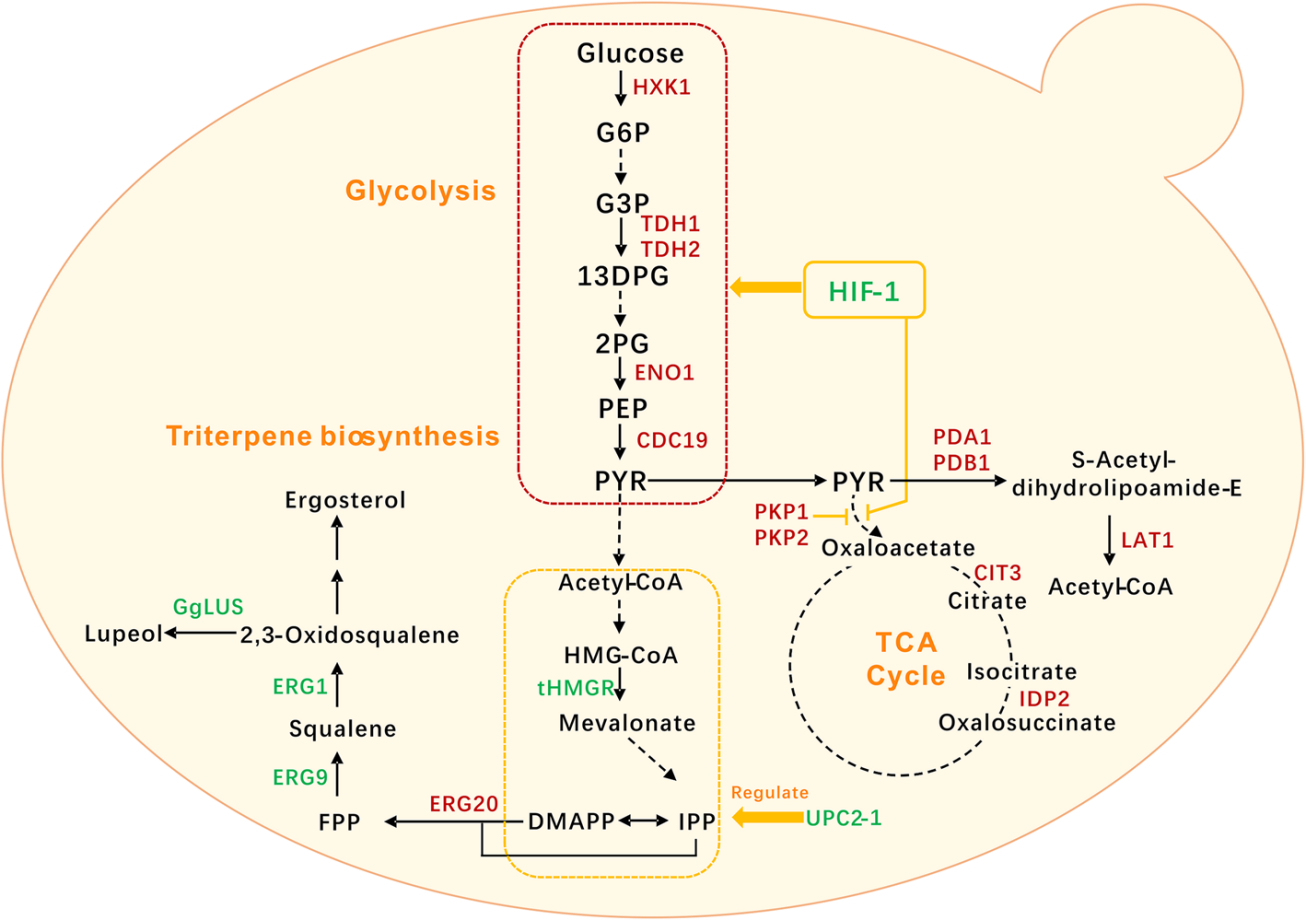
Overall strategy of HIF-complex-mediated CCM redirection for the biosynthesis of triterpenoids. G6P, glucose-6-phosphate; G3P, glyceraldehyde-3-phosphate; 13DPG, glyceraldehyde-1,3-phosphate; 2GF, Glycerate-2- phosphate; PEP, phosphoenolpyruvate; PYR, pyruvate; TCA Cycle, tricarboxylic acid cycle; HMG-CoA, 3-hydroxy-3-methyl-glutaryl-CoA; FPP, farnesyl pyrophosphate; HXK1, hexokinase 1; TDH1, glyceraldehyde-3-phosphate dehydrogenase, isozyme 1; TDH2, glyceraldehyde-3-phosphate dehydrogenase, isozyme 1; ENO1, enolase I; CDC19, pyruvate kinase; CIT3, citrate synthase; IDP2, isocitrate dehydrogenase 2; PDA1, pyruvate dehydrogenase alpha; PDA2 pyruvate dehydrogenase alpha; LAT1, dihydrolipoamide acetyltransferase component of the pyruvate dehydrogenase complex; PKP1, pyruvate dehydrogenase kinase gene 1; PKP2, pyruvate dehydrogenase kinase gene 2; tHMGR, truncated 3-hydroxy-3-methylglutaryl-CoA reductase; ERG20, farnesyl pyrophosphate synthetase;ERG9, squalene synthase; ERG1, squalene monooxygenase; HIF-1, hypoxia-inducible factor 1; UPC2-1, mutant of sterol uptake control protein 2. Overexpressed genes are shown in green, and orange arrows indicate positive regulation

In this work, we triggered the Warburg effect-like metabolic reprogramming of the CCM in yeast by overexpression of the human HIF-1 complex, composed of HIF-1α and ARNT. This rewiring of CCM significantly promoted the biosynthesis of ergosterol and lupeol to 1145.95 mg/L and 236.35 mg/L in shake flask cultivation, a 10.5-fold and 9.2-fold increase than strains without HIF-1 complex (Fig. 1). Transcriptome analysis reveals that glycolysis is enhanced while the tricarboxylic acid (TCA) cycle is attenuated in the HIF-1 complex overexpressed strain. Our study showed that the HIF-1 complex can be used as an efficient engineering strategy for the production of triterpenes in yeast.

## Results

### Overexpression of the HIF-1 complex improved the biosynthesis of squalene in yeast

**Fig. 2:**
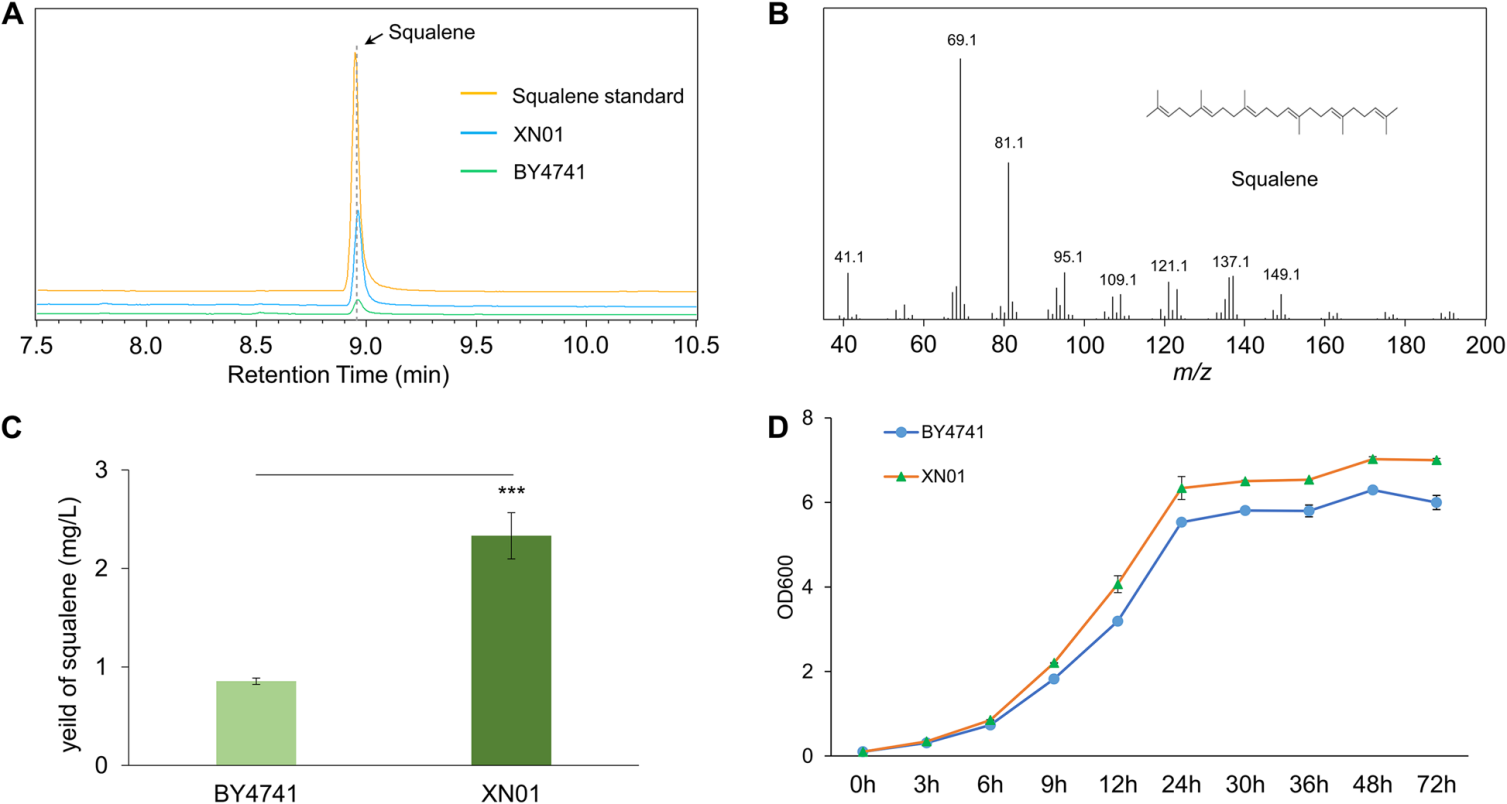
HIF-1 promoted the biosynthesis of squalene in yeast. **A** GC–MS analysis of cell extraction of BY4741 and strain XN01. **B** Mass spectra of squalene. **C** The yield of squalene in BY4741 and strain XN01. **D** The growth curve of BY4741 and strain XN01. Three repeats were performed for each strain, and the error bars represented the standard deviation

To trigger the similar Warburg effect with aerobic glycolysis of cancer cells in yeast, we integrated an expression cassette containing HIF-1α and ARNT genes, encoding the two subunits of HIF-1 complex, into the yeast chromosome of strain BY4741, deriving the strain XN01. Since squalene is the representative six-isoprenoid unit triterpene originating from acetyl-CoA in yeast, we use the content of squalene as the target chemical to evaluate the influence of the HIF-1 complex on cellular metabolism. After cultivation for 4 days using glucose as the carbon source, the production of squalene in strain XN01 and BY4741 was detected by GC–MS equipment. The GC profile and MS data compared with authentic squalene standard confirmed the production of squalene in both strains (Fig. 2A and B). According to the standard curve of squalene (Fig. S1A), the yield of squalene in strain XN01 was 2.33 mg/L, increased by 2.7-fold than that in BY4741 (0.85 mg/L) (Fig. 2C). This result revealed that ectopic expression of HIF-1 complex improved the biosynthesis of squalene in yeast.

As the aerobic glycolysis facilitated the proliferation rate of cancer cells, next, to observe the effect of HIF-1 complex induced aerobic glycolysis on the division of yeast cells, we monitored the growth of strain XN01 during cultivation. The growth curve demonstrated that strain XN01 exhibited a significantly higher cell concentration after 3 h of cultivation than BY4741, and this higher cell density lasted in exponential and stationary phases (Fig. 2D), which indicated that the HIF-1 complex conferred a strong growth condition to yeast cells. Then we compared the glucose consumption of strain XN01 and BY4741, and found that the glucose consumption of XN01 displayed a faster consume of glucose (Fig. S2), which might be responsible for the accelerated growth rate of strain XN01.

### Transcriptome analysis revealed that the HIF-1 complex redirected the metabolic flux from glycolysis to the TCA cycle in yeast

**Fig. 3:**
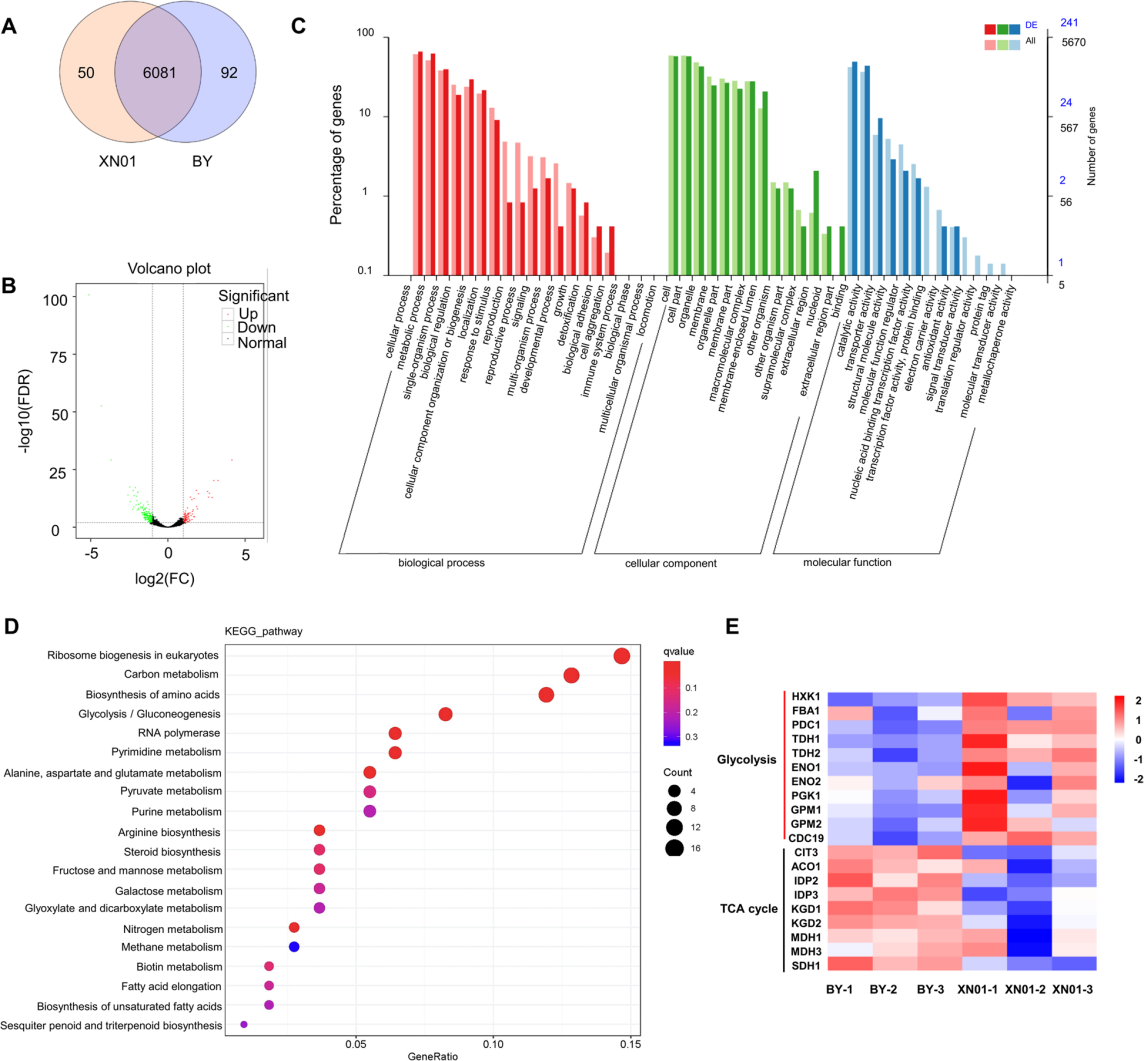
Transcriptome analysis of BY4741 and strain XN01. **A** Venn diagram of genes detected in the transcriptome data of BY4741 and strain XN01. **B** The volcanic map of DEGs between BY4741 and strain XN01. **C** GO enrichment of DEGs between BY4741 and strain XN01. **D** KEGG analysis of DEGs between BY4741 and strain XN01. **E** Heatmap of DEGs involved in glycolysis and TCA cycle between BY4741 and strain XN01

In cancer cells, the HIF-1 complex mediated Warburg effect was marked by the strengthened glycolysis and impaired metabolic flux into mitochondria (Lu et al. 2015). To detect whether the ectopic expression of the HIF-1 complex also caused similar metabolic reprogramming in yeast, we performed RNA sequencing using three biological replicates of enriched RNA samples from strains XN01 and BY4741. It was found that 50 and 92 genes were specifically expressed in strain XN01 and BY4741, respectively (Fig. 3A). As visualized in Fig. 3B, there were 263 differentially expressed genes (DGEs) in strain XN01, including 76 up-regulated genes and 187 down-regulated ones. The Gene Ontology (GO) enrichment analysis of DEGs showed that the enriched GO terms were significantly assigned to cellular process and metabolic process in the biological processes (BP) category, and binding and catalytic activity in the molecular function (MP) category (Fig. 3C). To get further insight into the biochemical functions of the DEGs, the Kyoto Encyclopedia of Genes and Genomes (KEGG) pathway enrichment analysis was performed. According to the KEGG enrichment (Fig. 3D), the majority of DEGs were significantly enriched in the ribosome, carbon metabolism, biosynthesis of amino acids, and glycolysis/gluconeogenesis. This enrichment is similar to what was observed in cancer cells ribosome and glycolysis/gluconeogenesis pathways were the most significant pathways in KEGG enrichment (Siavoshi et al. 2022).

Next, to explore the effect of the HIF-1 complex on the CCM of strain XN01, especially glycolysis and TCA cycle, we focused on the genes encoding enzymes in these metabolic pathways. Transcriptome data revealed that almost all the genes involved in glycolysis were upregulated, and particularly, the expression levels of HXK1, TDH1, TDH2, ENO1, and CDC19 were significantly increased with log2FoldChange > 1 (Fig. 3E). On the contrary, most genes encoding enzymes in TCA cycle were downregulated, such as CIT3 and IDP2 (Fig. 3E). The expression of selected genes in glycolysis and TCA cycle was verified by quantitative real-time PCR (qRT-PCR) (Fig. S3). These results indicated that HIF-1 complex systematically redirected the metabolic flux from glycolysis to TCA cycle in yeast, which was consistent with the HIF-1 induced Warburg effect in cancer cells, implying the conserved function of HIF-1 complex in human and yeast.

### Optimization of MVA and steroid synthesis pathways further promoted the production of squalene in HIF-1 overexpressed yeast strains

In yeast MVA pathway, the 3-hydroxy-3-methylglutaryl-CoA reductase (HMGR), encoded by the HMG1 gene, is the major rate-limiting enzyme, and overexpression of the catalytic domain of HMG1 (truncated HMG1) led to improved production of isoprenoid units, isopentenyl pyrophosphate (IPP) and dimethylallyl pyrophosphate (DMAPP) (Donald et al. 1997; Hampton et al. 1996; Polakowski et al. 1998). In the subsequent steroid synthesis pathway, the isopentenyl transferase ERG20 condenses IPP and DMAPP to form the linear isopentenyl diphosphate precursor, farnesyl diphosphate (FPP). Then squalene synthase ERG9 converts FPP to squalene. Optimization of these two pathways is the generally used strategy to increase the production of triterpenes in engineered yeast strains (Dai et al. 2012). Meanwhile, UPC2 is transcription factor, belonging to the C6 zinc finger class, and regulates a number of ERG genes in the yeast ergosterol biosynthetic pathway (Yang et al. 2015). Overexpression of The UPC2-1 variant, containing a single amino acid mutation (G888D) within the activation domain (MacPherson et al. 2006), exerts modest effects on terpenoids biosynthesis in yeast (Peralta-Yahya et al. 2011; Ro et al. 2006).

**Fig. 4:**
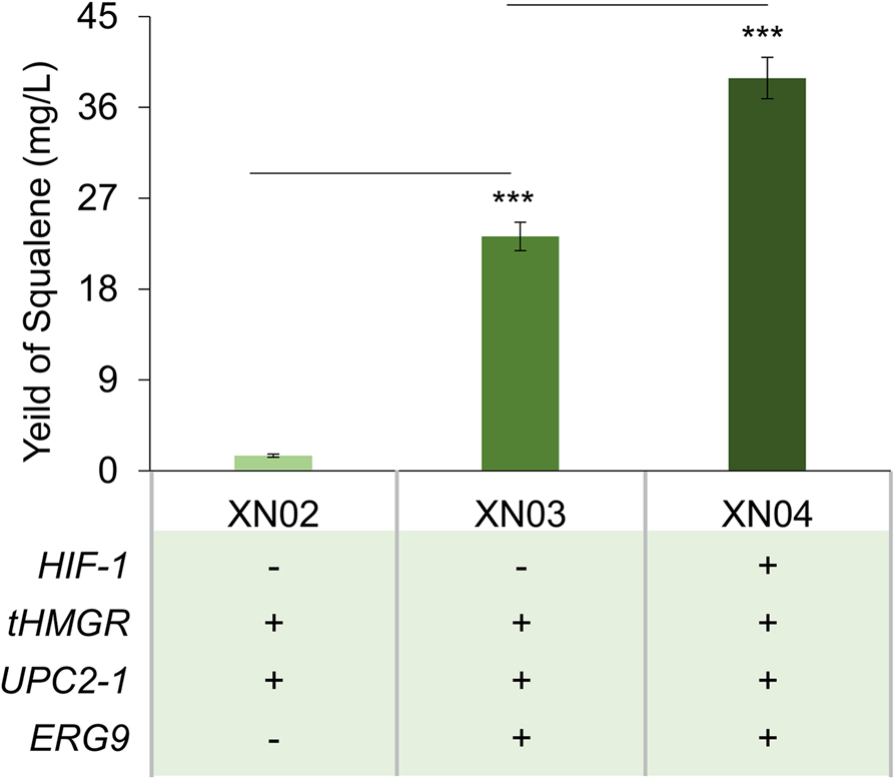
The production of squalene in different strains

Therefore, to further increase the production of squalene, we integrated a single copy of an expression cassette containing the truncated HMG1 (tHMGR) and a variant of UPC2 (UPC2-1), the global regulatory factor MVA pathway, into the chromosome of BY4741, generating strains XN02. GC–MS analysis showed that the production of squalene in strain XN02 was 1.51 mg/L, 1.8-fold higher than that of BY4741 (Figs. 2C and 4). Notably, the production of squalene in XN01 with HIF-1 complex was much higher than this MVA pathway optimized strain XN02, about 1.5-fold increase (Figs. 2C and 4). Next, to largely enhance the biosynthesis of squalene, we integrated multiple copies of the above expression cassette into the TY4 sites of the genome of BY4741 and XN01, respectively, and then ERG9 of both subsequent strains was also overexpressed by replacing its native promoter with galactose-induced promoter GAL10p, getting strains XN03 and XN04. Compared to strain XN03, the introduction of the HIF-1 complex in XN04 promoted the production of squalene to 38.90 mg/L, which increased by 1.7-fold higher (Fig. 4). These results indicated that manipulation of the HIF-1 complex was efficient for squalene production in engineered yeast strains.

### HIF-1 complex boosted the biosynthesis of endogenous ergosterol in yeast

**Fig. 5:**
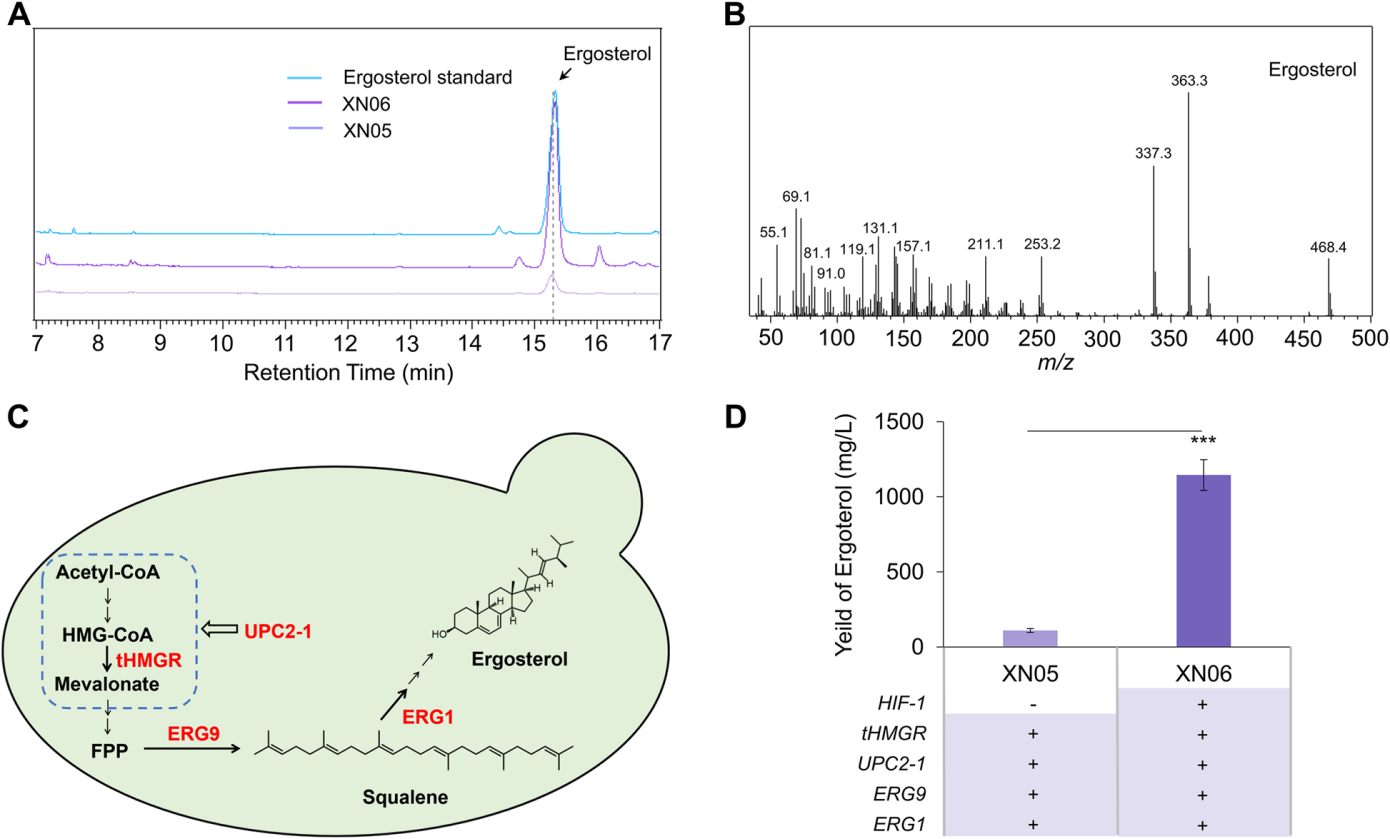
HIF-1 boosted the production of ergosterol in engineered yeast strains. **A** GC–MS analysis of cell extraction of strains XN05 and XN06. **B** Mass spectra of ergosterol. **C** The optimization strategy for the biosynthesis of ergosterol. **D** The production of ergosterol in strain XN05 and XN06

As the HIF-1 complex promoted the production of squalene, the precursor of triterpenes and sterols in yeast, next, to evaluate the promotion effect of the HIF-1 complex on the biosynthesis of triterpenoids, we first selected ergosterol (the major sterol in yeast) as the representative endogenous target. We detected the ergosterol content in XN01 (Fig. 5A-B), and the result showed that the production of ergosterol in this strain was 0.18 mg/L, which was 2.5-fold higher than that in BY4741 according to the standard curve of the authentic ergosterol (Figs. S1B and Fig. S4).

To further promote the metabolic flux towards the synthesis of ergosterol, we overexpressed squalene epoxide gene ERG1 by replacing its native promoter with galactose-induced promoter GAL10p in strains XN03 and XN04, getting strains XN05 and XN06 (Fig. 5C). The quantitative analysis showed that strain XN06 produced 1145.95 mg/L ergosterol, with 10.5-fold higher than strain XN05 (Fig. 5D).

### HIF-1 complex significantly improved the biosynthesis of exogenous triterpene in yeast

**Fig. 6:**
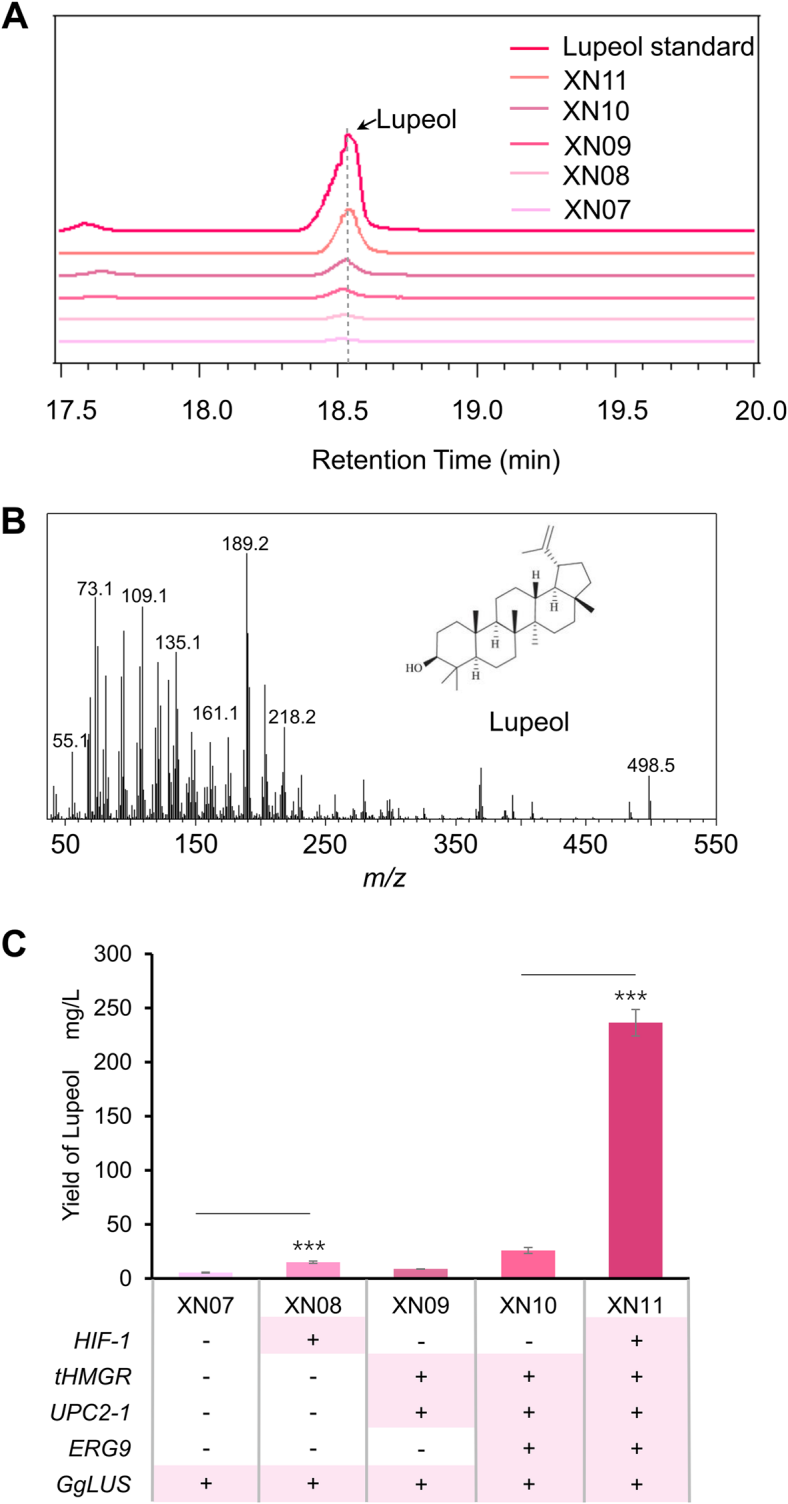
HIF-1 boosted the production of lupeol in engineered yeast strains. **A** GC–MS analysis of cell extraction in different strains. **B** Mass spectra of lupeol. **C** The production of lupeol in different strains

Next, to make clear whether the HIF-1 complex could facilitate the biosynthesis of exogenous triterpene, lupeol was selected as the represent triterpene, and the lupeol synthase GgLUS from *Glycyrrhiza glabra* was used here to produce lupeol in engineered yeast. The expression vector containing GgLUS was transformed into BY4741 and strains XN01-XN04, to get strains XN07-XN11. Then the shake flask cultivation was performed and the production of lupeol was confirmed by the comparison of retention time and MS profile with authentic lupeol (Fig. 6A-B). The yield of lupeol was calculated according to the standard curve of the authentic lupeol (Fig. S1C). Results showed that strain with HIF-1 complex brought about remarkable promotion in the biosynthesis of lupeol. Strain XN08 produced 14.86 mg/L lupeol, 2.8-fold higher than that in BY4741 (Fig. 6C). Notably, the production of lupeol in this strain was much higher than in strain XN09 (8.65 mg/L) with one copy of tHMGR and UPC2-1 overexpression cassette in the chromosome (Fig. 6C). Even more surprisingly, the HIF-1 complex boosted the lupeol production to 236.35 mg/L in highly optimized strain XN11, a 9.2-fold increase compared to strain XN10 without HIF-1 transformation (Fig. 6C). These results collectively indicated that the HIF-1 complex improved the yield of squalene-derived triterpenoids, especially boosting their output in highly engineered yeast strains.

## Discussion

CCM is the basic energy metabolism inside cells and it contains three metabolic pathways, including glycolysis, the TCA cycle, and the pentose phosphate pathway (PPP). The previous optimization of CCM in engineered yeast was mainly focused on the individual enzymes in CCM to rewire the metabolic flux among the above three pathways. These methods have been successfully used in the microbial production of aromatic amino acids and isoprenoids and significantly promoted the output of these chemicals (Hassing et al. 2019; Liu et al. 2019; Meadows et al. 2016). In the present study, a new strategy was developed to regulate the metabolic flux of CCM without the manipulation of enzymes in this energy metabolism. We introduced the HIF-1 complex (HIF-1α and ARNT) into the engineered yeast strains to fulfill enhanced glycolysis and attenuated TCA cycle. This redirection of metabolic flux resulted in a marked increase in the production of triterpenoids.

In cancer cells, the HIF-1 complex plays a key role in the reprogramming of glycolysis. The hypoxia condition promotes the accumulation of subunits of the HIF-1 complex and these proteins further increase the expression levels of genes encoding enzymes in glycolysis, such as HXK2, GAPDH, and PKM2 (Porporato et al. 2011). In the meanwhile, the HIF-1 complex activates the enzyme pyruvate dehydrogenase kinase isozyme 1 (PDK1), which prevents the entry of pyruvate into the TCA cycle (Papandreou et al. 2006; Semba et al. 2016). Consistent with the regulation of HIF-1 to glycolysis, the result here showed that the introduction of the HIF-1 complex into yeast enhanced the glycolysis pathway by upregulating the expression of the glycolytic genes (Fig. 3E). As the flow of pyruvate into TCA cycle is inhibited by PDK1 in human cells, we checked the expression of pyruvate dehydrogenase kinase in strain XN01. The transcriptome analysis and qRT-PCR verification revealed that the two pyruvate dehydrogenase kinase genes PKP1 and PKP2 responsible for the negative regulation of yeast pyruvate dehydrogenase complex were both slightly activated, and PKP2 in particular, was also showed increased significant expression level determined by qPCR (Fig. S5). These results indicated the conserved regulation roles of the HIF-1 complex to CCM in mammals and yeast.

The engineering of the MVA pathway is the commonly used method for the microbial production of terpenoids, and the optimization of tHMGR and UPC2-1 is very effective. Meanwhile, the newly developed metabolic strategies in recent years have greatly improved the production of triterpenoids, such as increasing endoplasmic reticulum (ER) expansion (Arendt et al. 2017; Kim et al. 2019). This strategy usually enhanced the yield of targeted triterpenoids to about 6–eightfold higher in highly engineered strains. Here in our study, the transformation of the HIF-1 complex into the basic strain BY4741 produced more squalene and lupeol than BY4741 with tHMGR and UPC2-1 integration (Figs.4 and 6C). More excitedly, the promotion of HIF-1 complex to the biosynthesis of triterpenoids was strongly displayed in highly engineered strains. HIF-1 increased the production of ergosterol and lupeol to 1145.95 mg/L and 236.35 mg/L, 10.5-fold and 9.2-fold higher than that in strain XN05 and XN10 in shake flask cultivation, respectively (Figs. 5D and 6C). Therefore, the manipulation of HIF-1 is a promising engineered method for the biosynthesis of triterpenoid in yeast cells.

The pyruvate dehydrogenase bypass (PDB) is responsible for the catalyzation of pyruvate to acetyl-CoA in yeast cytosol (Shiba et al. 2007). According to our transcriptome data, the expression levels of genes encoding enzymes in this bypass, including PDA1, PDB1, and LAT1, had no significant differences between BY4741 and strain XN01 or the expression of these genes were slightly downregulated in strain XN01 revealed by qRT-PCR (Fig. S6). This signified that the accumulated pyruvate in the cytosol of strain XN01 induced by HIF-1 complex mediated-CCM redirection was not effectively transformed to acetyl-CoA. Therefore, enhancing the PDB would further increase the production of acetyl-CoA and subsequently promote the biosynthesis of triterpene in strains with the HIF-1 complex. Meanwhile, in addition to MVA pathway, acetyl-CoA can enter multiple pathways, including fatty acids biosynthesis, glyoxylate cycle and chromatin acetylation (Nielsen 2014) and the increased acetyl-CoA would bring about positive effect to these pathways, for example, promoting the biosynthesis of fatty acids. Furthermore, acetyl-CoA serves as a particularly important precursor for many other different chemical products, such as polyhydroxybutyrates (PHB), polyketides and fatty acyl ethyl esters (de Jong et al. 2014; Kocharin et al. 2012; Zhao et al. 2020), thus the enhanced acetyl-CoA flux caused by HIF-1 complex could be successively engineered for the biosynthesis of these valuable acetyl-CoA-derived products in yeast cell factories.

## Conclusions

In this study, we improved the biosynthesis of squalene by overexpression of the human HIF-1 complex to enhance glycolysis and redirect the metabolic flux from glycolysis to the TCA cycle. The introduction of HIF-1 significantly increased the production of squalene and squalene-derived triterpenoids in engineered yeast strains. Interestingly, the promotion of the HIF-1 complex to the biosynthesis of triterpenoids was strongly displayed in highly engineered strains. Therefore, the HIF-1 complex can be used as an efficient engineering strategy for the high-level production of triterpenes in yeast.

## Materials and methods

### Strains, medium, and reagents

The yeast strain used in this work is all derived from BY4741. E. coli Trans5α chemically competent cell (TransGen Biotech, Beijing, China) was used for plasmid construction. Luria–Bertani (LB) broth with corresponding antibiotics was used for the cultivation of recombinant E. coli. YPD (1% yeast extract, 2% peptone, and 2% glucose) was used for the cultivation of yeast. Synthetic dropout (SD) medium and drop-out medium (synthetic complete drop-out medium with 2% carbon source) were used for the selection and cultivation of yeast strains. All restriction enzymes were purchased from Thermo Fisher (Thermo Fisher Scientific, USA). ClonExpress MultiS One Step Cloning Kit was purchased from Vazyme Biotech (Nanjing, China). PrimeSTAR Max DNA Polymerase was purchased from Takara (Shiga, Japan). Primers were synthesized by Sangon Biotech (Shanghai, China). Frozen-EZ Yeast Transformation II Kit was purchased from ZYMO Research (California, USA). The standard squalene and ergosterol were purchased from Shanghai Yuanye Bio-Technology (Shanghai, China).

### Plasmids and strains construction

To construct cassettes of all the expression cassettes, four empty vectors (pTP-URA, pESC-LEU, pESC-URA, pCfB2796, pEASY-Blunt Simple) were used in this study. All the fragments used for gene expression were amplified by PCR method using primers listed in Supplementary Table. Genes HIF-1α (GenBank accession number NM_001243084.2) and ARNT (GenBank accession number NM_001243084.2) were codon-optimized and synthesized by Sangon Biotech (Shanghai, China). Plasmids were constructed by the in-fusion cloning method. The expression cassettes were transformed into yeast by Frozen-EZ Yeast Transformation II Kit. All the plasmids and strains constructed in this study were listed in Table 1, and primers used for these constructions were listed in Table S1.

**Table 1:** Plasmids and strains constructed in this study

**Plasmids**	**Description**	**Source**
pESC-URA	Yeast expression vector with URA selection maker	Stored in the lab
pESC-LEU	Yeast expression vector with LEU selection maker	Stored in the lab
pESC-MET	Yeast expression vector with MET selection maker	
pCfB2796	Yeast expression vector with TY4 integration sites and URA selection maker	Stored in the lab
pEASY-Blunt Simple	Cloning vector	Stored in the lab
pTP-URA	Replace the GAL10p and GAL1p of pESC-URA to PGK1p and TEF1p	This study
pTP-URA-HIF-1α-ARNT	Insert HIF-1α and ARNT to the two multiple cloning sites of pTR-URA	This study
pESC-URA-tHMGR-UPC2-1	Insert tHMGR and UPC2-1 to the two multiple cloning sites of pESC-URA	This study
pCfB2796-tHMGR-UPC2-1	Insert the expression cassette containing tHMGR and UPC2-1 to pCfB2796	This study
pESC-MET-ERG9	Insert ERG9 to pESC-MET	This study
pESC-LEU-GgLUS	Insert GgLUS to pESC-LEU	This study
**Strains**	**Description**	**Source**
BY4741	MATα *HIS3Δ1 LEU2Δ0 MET15Δ0 URA3Δ0*	Stored in the lab
XN01	BY4741 NDT80::URA3-PGK1p-HIF-1α-CYC1t-TEF1p-ARNT-ADH1t	This study
XN02	BY4741 NDT80::URA3-GAL1p-tHMGR-CYC1t-GAL10p-UPC2-1-ADH1t	This study
XN03	BY4741 TY4::URA3/GAL1p-tHMGR-CYC1t-GAL10p-UPC2-1-ADH1t; ERG9p::HIS-GAL10p	This study
XN04	XN01 TY4::URA3/GAL1p-tHMGR-CYC1t-GAL10p-UPC2-1-ADH1t; ERG9p::HIS-GAL10p	This study
XN05	XN03 ERG1p::MET-GAL1p-ERG9-GAL10p	This study
XN06	XN04 ERG1p:: MET-GAL1p-ERG9-GAL10p	This study
XN07	BY4741 pESC-LEU-GgLUS	This study
XN08	XN01 pESC-LEU-GgLUS	This study
XN09	XN02 pESC-LEU-GgLUS	This study
XN10	XN03 pESC-LEU-GgLUS	This study
XN11	XN04 pESC-LEU-GgLUS	This study

### Shake flask cultivation

SD medium with corresponding amino acids and carbon source was used for the cultivation of the engineered yeast strains. Single colonies of all strains were firstly inoculated into 12 mL test tubes containing 5 mL medium, and grown to the OD600 of 0.6–0.8 at 30℃ and 200 rpm. Then flasks (250 mL) containing 50 mL medium were then inoculated with these seed cultures at an initial OD600 of 0.1. The cultures were grown at 30℃ and 200 rpm for 4–6 days. All the flask fermentation results were represented as the means ± S.D. from three independent replicates.

### Extraction and analytical methods

Yeast cells were harvested by centrifugation at 5000 × g for 5 min. The harvested cells were then resuspended in 10 mL of 1:1 ethanol-potassium hydroxide (EP) solution and boiled for 10 min. Squalene and ergosterol were extracted with an equal volume of hexane. After filtering by 0.22 μm syringe filters, the metabolites were analyzed using an Agilent gas chromatograph-mass spectrometer (GC–MS) autosampler.

The GC–MS system (Agilent 7693A, USA) was equipped with an HP-5 column and a flame ionization detector (FID). The oven temperature was first maintained at 160℃ for 1 min, followed by gradually increasing to 280 °C at a rate of 30 °C / min and hold for 10 min. Finally, increased to 300 °C at a rate of 2 °C / min and hold for 5 min. All results were presented as the average of three replicates.

### Glucose consumption detection

The glucose concentration in the medium was detected by Glucose Detection Kit (Qiyuan Bio) according to the instruction. Briefly, add 50 μL of the Reaction Mix to the wells containing the Glucose Standard and test samples, and mix well. Incubate the reaction for 30 min in dark. Then measure absorbance of the solution at 505 nm, and calculate the glucose concentration according to the standard curve.

### RNA-sequencing

After 24 h of cultivation, the cells were harvested by centrifugation. RNA was extracted using the TRIzol method following the manufacturer’s protocol. The integrity of the RNA was examined by the RNA integrity number (RIN) using an Agilent 2100 bioanalyzer (Agilent, Santa Clara, USA). Oligo(dT)-attached magnetic beads were used to purify mRNA. Using mRNA as a template, the first strand of cDNA was synthesized with random hexamers, and then the second cDNA chain was synthesized by adding buffer, dNTPs, RNase H, and DNA polymerase I, and the cDNA was purified using AMPure XP beads. The purified double-stranded cDNA was repaired at the end, a-tails were added, and sequencing joints were connected. Then AMPure XP Beads were used for fragment size selection, and cDNA libraries were enriched by PCR. After the library is qualified, different libraries were pooled according to the target data volume and sequenced on the Illumina platform (BIOMARKER, China). The False Discovery Rate (FDR) was obtained by correcting for the *p*-value of the significance of the difference. Fold Change (FC) represented the ratio of expression between two groups. During the detection of differentially expressed genes, FC ≥ 2 and FDR < 0.01 were used as screening criteria.

### qRT-PCR

Total RNAs of the yeast cells were isolated by Yeast Total RNA Isolation Kit (Sangon). 1 μg total RNA was used for cDNA synthesis and the qRT-PCR was performed on LightCycler 96 (Roche). The PCR conditions included an initial denaturation step of 95 °C for 3 min and 40 cycles of 95 °C for 10 s, 60 °C for 10 s. Primers used for qRT-PCR were listed in Table S2.

## Supplementary Information

**Additional file 1: Fig. S1.** The external calibration curves of different chemicals. The external calibration curves of squalene (a), ergosterol (b), and lupeol (c). **Fig. S2.** The glucose consumption of strain XN01 and BY4741. **Fig. S3.** The expression levels of genes involved in glycolysis and TCA cycle from the transcriptome data and qRT-PCR. **Fig. S4.** The production of ergosterol in BY4741 and strain XN01. Three repeats were performed for each strain, and the error bars represented the standard deviation. **Fig. S5.** The expression level of PKP1 and PKP2 in BY4741 and strain XN01 from the transcriptome data and qRT-PCR. **Fig. S6.** The expression level of PDA1, PDB1, and LAT1 in BY4741 and strain XN01 from the transcriptome data and qRT-PCR. **Table S1.** Primers used in plasmids and strains construction. **Table S2.** Primers used for qRT-PCR.

## Data Availability

The RNA-seq raw data has been deposited in NCBI database with the NCBI Sequence Read Archive (SRA) database under the accession code PRJNA1018926. All data generated or analyzed during this study are included in this published article and its supplementary information file.
