# Supplementary material for: Optimization of central carbon metabolism by Warburg effect of human cancer cell improves triterpenes biosynthesis in yeast
Source: Adv Biotechnol (Singap). 2023 Oct 26;1(4):4. doi: 10.1007/s44307-023-00004-6 (PMC11727583; doi:10.1007/s44307-023-00004-6)
Supplement: Supplementary file 1 — Additional file 1: Fig. S1. The external calibration curves of different chemicals. The external calibration curves of squalene (a), ergosterol (b), and lupeol (c). Fig. S2. The glucose consumption of strain XN01 and BY4741. Fig. S3. The expression levels of genes involved in glycolysis and TCA cycle from the transcriptome data and qRT-PCR. Fig. S4. The production of ergosterol in BY4741 and strain XN01. Three repeats were performed for each strain, and the error bars represented the standard deviation. Fig. S5. The expression level of PKP1 and PKP2 in BY4741 and strain XN01 from the transcriptome data and qRT-PCR. Fig. S6. The expression level of PDA1, PDB1, and LAT1 in BY4741 and strain XN01 from the transcriptome data and qRT-PCR. Table S1. Primers used in plasmids and strains construction. Table S2. Primers used for qRT-PCR. [file 44307_2023_4_MOESM1_ESM.docx]

**Supplementary Information**

**Optimization of central carbon metabolism by Warburg effect of human cancer cell improves triterpenes biosynthesis in yeast**

Xiaona Lin^1,#^, Tianyue An^2,#^, Danni Fu^1,#^, Sujuan Duan^1^, Hong-Lei Jin^1,3,4*^ and Hong-Bin Wang^1,5,6,*^

^1^Institute of Medical Plant Physiology and Ecology, School of Pharmaceutical Sciences, Guangzhou University of Chinese Medicine, Guangzhou 510006, China

^2^School of Integrated Traditional Chinese and Western Medicine, Binzhou Medical University, Yantai 264003, China

^3^Guangzhou Key Laboratory of Chinese Medicine Research on Prevention and Treatment of Osteoporosis, The Third Affiliated Hospital of Guangzhou University of Chinese Medicine, Guangzhou 510006, China

^4^Key Laboratory of Chinese Medicinal Resource from Lingnan (Guangzhou University of Chinese Medicine), Ministry of Education, Guangzhou 510006, China

^5^Key Laboratory of Chinese Medicinal Resource from Lingnan (Guangzhou University of Chinese Medicine), Ministry of Education, Guangzhou 510006, China

^6^State Key Laboratory of Dampness Syndrome of Chinese Medicine, Guangzhou University of Chinese Medicine, Guangzhou 510006, China

^#^These authors contributed equally to this work.

* Corresponding authors. *E-mail addresses*: [jinhl@gzucm.edu.cn](mailto:jinhl@gzucm.edu.cn); [wanghongbin@gzucm.edu.cn](mailto:wanghongbin@gzucm.edu.cn)


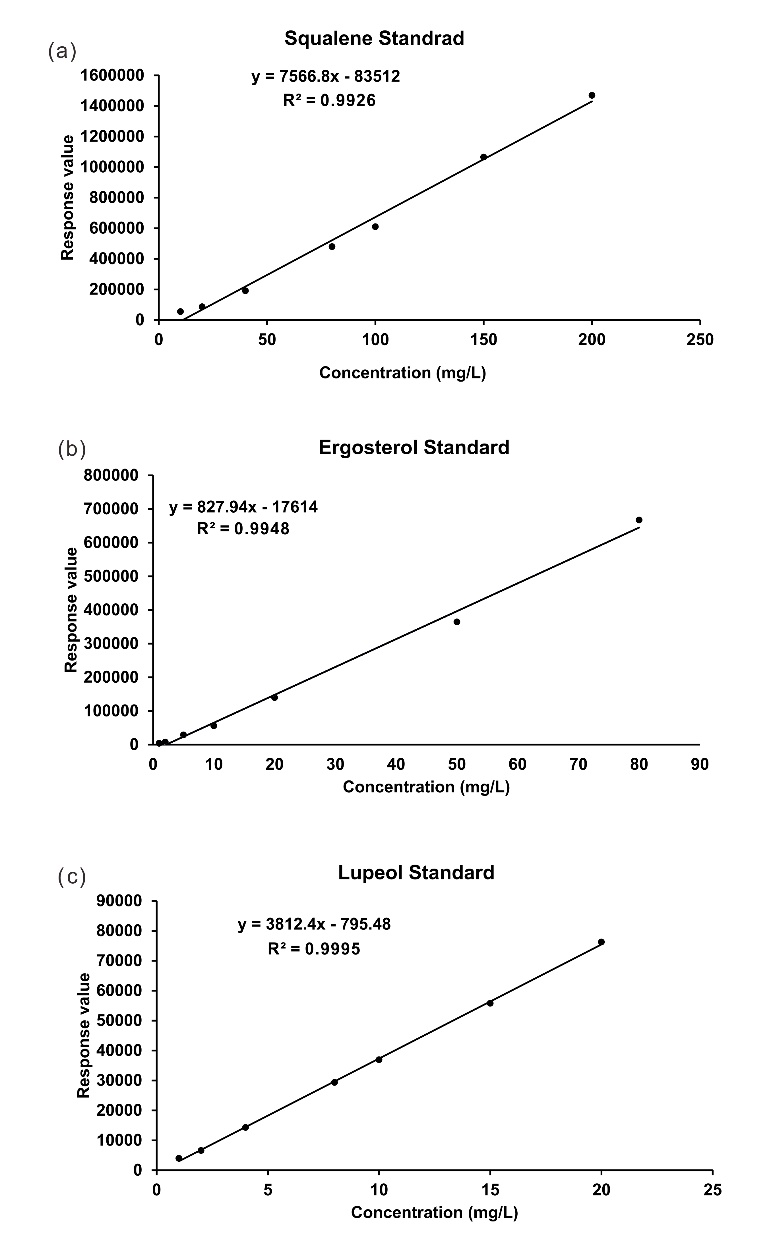


Fig. S1 The external calibration curves of different chemicals. The external calibration curves of squalene (a), ergosterol (b), and lupeol (c).


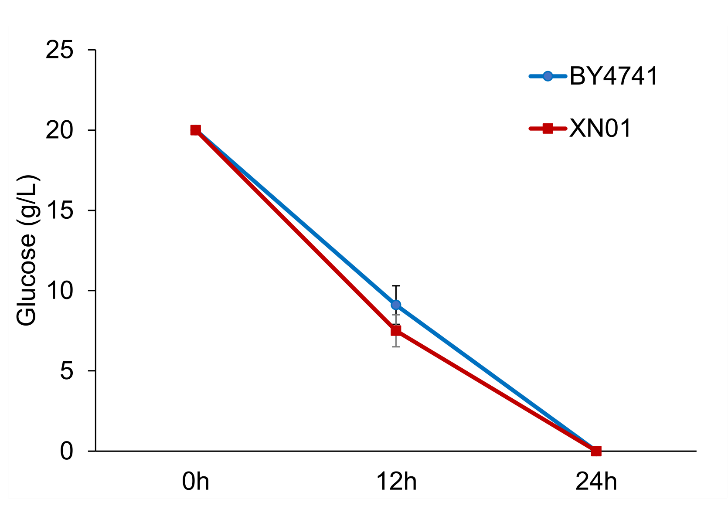


Fig. S2 The glucose consumption of strain XN01 and BY4741.


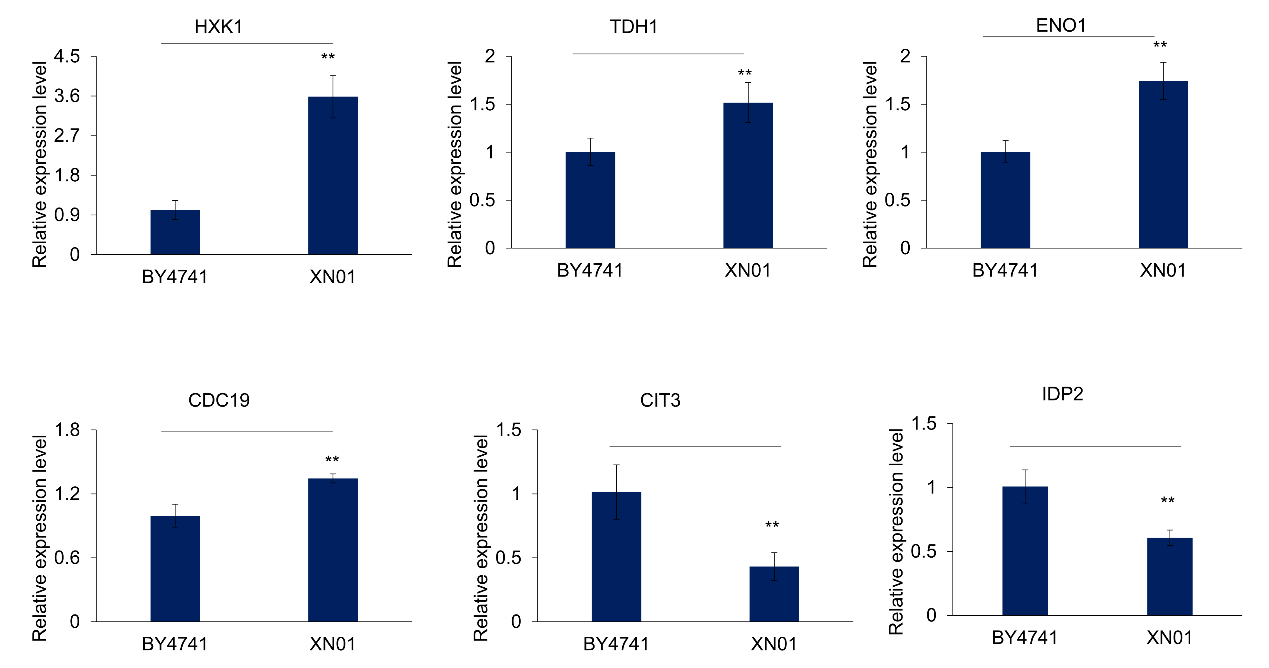


Fig. S3 The expression levels of genes involved in glycolysis and TCA cycle from the transcriptome data and qRT-PCR.


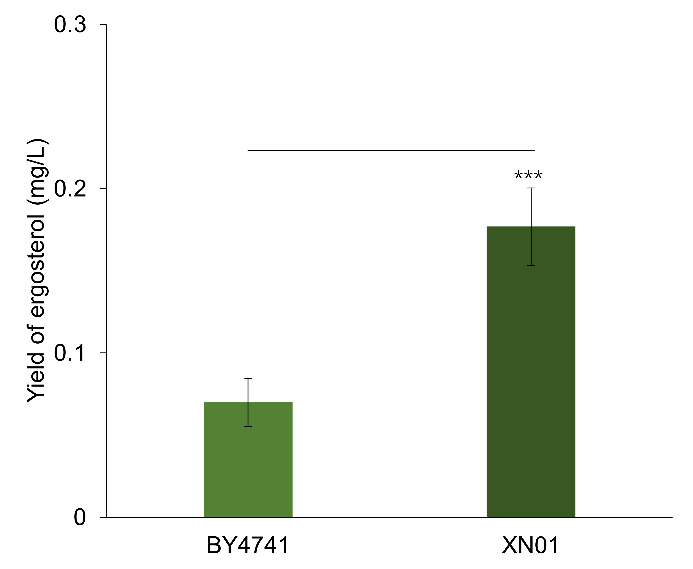


Fig. S4 The production of ergosterol in BY4741 and strain XN01. Three repeats were performed for each strain, and the error bars represented the standard deviation.


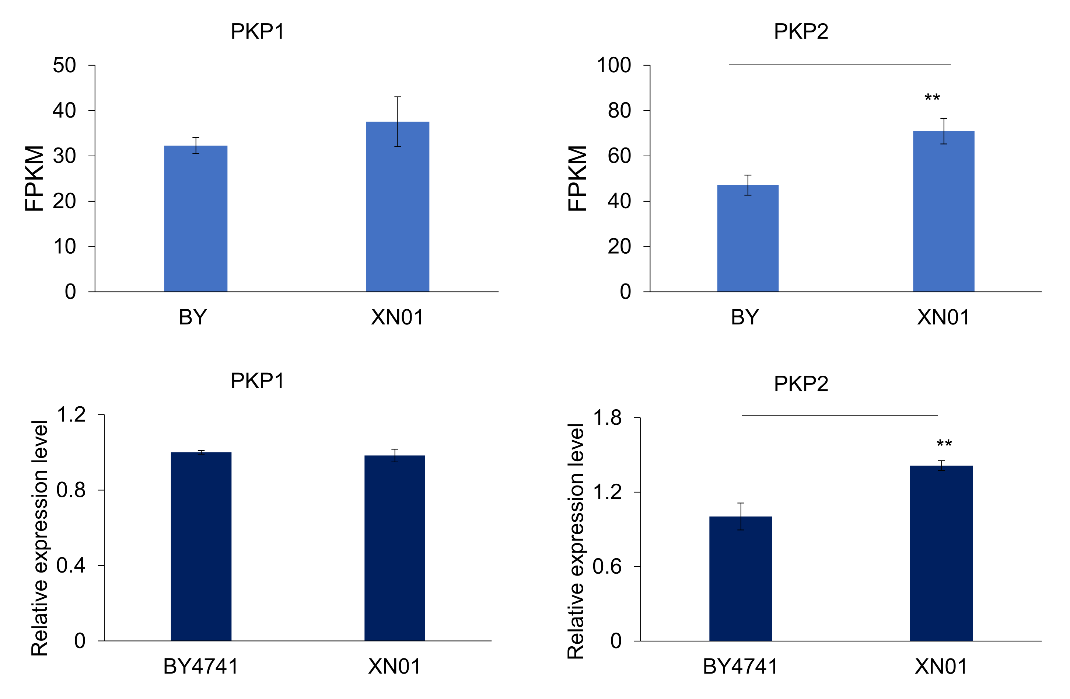


Fig. S5 The expression level of PKP1 and PKP2 in BY4741 and strain XN01 from the transcriptome data and qRT-PCR.


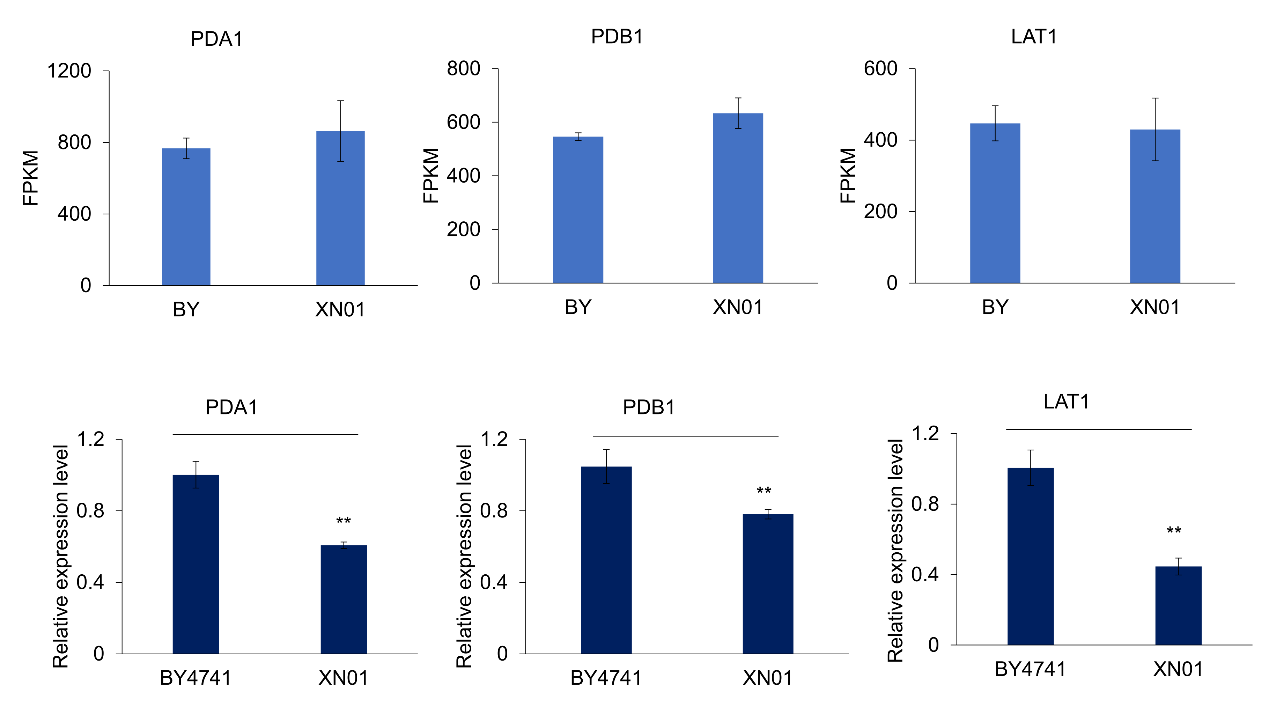


Fig. S6 The expression level of PDA1, PDB1, and LAT1 in BY4741 and strain XN01 from the transcriptome data and qRT-PCR.

Table S1 Primers used in plasmids and strains construction.

| **Primer name** | **Sequence 5’→3’** |
| --- | --- |
| pTR-HIF-1α-NotI-F | CAACAAATATAAAACAAGCGGCCGCATGAGCTCCCAATGTCGGAGTTTG |
| pTR-HIF-1α-SacI-R | GAAGAATTGTTAATTAAGAGCTCTCAGTTAACTTGATCCAAAGCTCTGAGT |
| pTR-ARNT-BamHI-F | CTAAGTTTTAATTACAAGGATCCATGGCGGCGACTACTGCCA |
| pTR-ARNT-NheI-R | GTTAGAGCGGATCTTAGCTAGCCTATTCTGAAAAGGGGGGAAACATAGTTA |
| NDT80-HIF-DF | ATGAATGAAATGGAAAACACAGATCCAGTATTACAGGGTGTCGGGGCTGGCTTAACTAT |
| NDT80-HIF-DR | TTAATACTTATAGAAACTATCTTCCTCAAACAATTCCTTCGAGCGTCCCAAAACCTTCT |
| pTR-tHMGR-NotI-F | CAACAAATATAAAACAAGCGGCCGCATGGCTGCAGACCAATTGGTGAA |
| pTR-tHMGR-SacI-R | GAAGAATTGTTAATTAAGAGCTCTCAGTTAGGATTTAATGCAGGTGACGGACC |
| pTR-UPC2-1-BamHI-F | CTAAGTTTTAATTACAAGGATCCATGAGCGAAGTCGGTATACAGAATCACA |
| pTR-UPC2-1-NheI-R | GTTAGAGCGGATCTTAGCTAGCTCATAACGAAAAATCAGAGAAATTTGTTGTTG |
| NDT80-tHMGR-UPC2-1-DF | ATGAATGAAATGGAAAACACAGATCCAGTATTACAGGGTGTCGGGGCTGGCTTAACTAT |
| NDT80-tHMGR-UPC2-1-DR | TTAATACTTATAGAAACTATCTTCCTCAAACAATTCCTTCGAGCGTCCCAAAACCTTCT |
| pESC-tHMGR-BcuI-F | CTAAAGGGCGGCCGCACTAGTATGGCTGCAGACCAATTGGTGAAAACTGAAG |
| pESC-tHMGR-SacI-R | AGAATTGTTAATTAAGAGCTCTTAGGATTTAATGCAGGTGACGGACCCATCT |
| pESC-UPC2-1-BamHI-F | AGGAGAAAAAACCCCGGATCCATGAGCGAAGTCGGTATACAGAATCACAAG |
| pESC-UPC2-1-NheI-R | GTTAGAGCGGATCTTAGCTAGCTCATAACGAAAAATCAGAGAAATTTGTTGTTGT |
| 2796-NheI-F | AGTTGAACATGTTATGCTAGCGAGCGACCTCATGCTATACCTGAGAAAGC |
| 2796-HindIII-R | GACCTGCAGCGTACGAAGCTTCTTCGAGCGTCCCAAAACCTTCTCAAGCAAGG |
| TY4-F | GGAACGAGAGTAATTAATAGTGACATGAG |
| TY4-R | CCGCGGCCGCAGATCTTGTT |
| ERG9oe-F | TTTGGGTTTAGTGCCTAAACGAGCAGCGAGAACACTCGCGCGTTTCGGTGATGACGGTG |
| ERG9oe-R | CGGATGCAATGCCAATTGTAATAGCTTTCCCATGGGTTTTTTCTCCTTGACGTTAAAGT |
| pESC-ERG1-EcoRI-F | AATTTTTGAAAATTCGAATTCATGTCTGCTGTTAACGTTGCACCTGAAT |
| pESC-ERG1-EcoRI-R | GAATTGTTAATTAAGAGCTCTTAACCAATCAACTCACCAAACAAAAATGG |
| ERG9oe-F-2 | TTTGGGTTTAGTGCCTAAACGAGCAGCGAGAACACCTTAACTATGCGGCATCAGAG |
| ERG9oe-R-2 | CGGATGCAATGCCAATTGTAATAGCTTTCCCATGGGTTTTTTCTCCTTGACGTTAAAGT |
| pESC-GgLUS-NotI-F | AACCCTCACTAAAGGGCGGCCGCATGTGGAAGCTGAAGATAG |
| pESC-GgLUS-SacI-R | AGAATTGTTAATTAAGAGCTCTCAATAACTGTGAGCACACAAGA |

Table S2 Primers used for qRT-PCR.

| **Primer name** | **Sequence 5’→3’** |
| --- | --- |
| qHXK1-F | CCGCTGACGGTTCTGTCTATAACAAATACC |
| qHXK1-R | CTGCACCTGAACCATCCTCAGCTG |
| qTDH1-F | CGGTAAGGTCTTGCCAGAATTGCA |
| qTDH1-R | CGGCAGCCTTAACAGCCTTCTTG |
| qTDH2-F | CAAGAAGGTTGTCAAGGCTGCCG |
| qTDH2-R | GATACCAGCGGCAGCATCGAAGAT |
| qENO1-F | CGGTACCTTGTCTGAATCCATCAAGGC |
| qENO1-R | GTCTTGATTTGACCAGTTCTCAAACCGA |
| qCDC19-F | GACCAAACTGTCCAATCATCTTGGTTACC |
| qCDC19-R | CCGAAGTTGATACGGGCTTCAACAT |
| qCIT3-F | TTCCAGCATTGCACGAGAACAAGAA |
| qCIT3-R | TTGCGCAAATTCAAGCATTGCTGTA |
| qIDP2-F | CGACGGTGACGTAGAATCAGATATTGTTG |
| qIDP2-R | AATGTCTTGTTACTGTACCGTGGGCG |
| qPDA1-F | TCAAGATCCTCCGCGATGACTGAATAT |
| qPDA1-R | TAGAACGAGAGGACCTTTGCCGGAT |
| qPDB1-F | GTGTCTCTGCAGAAGTTATCAACTTGCGTT |
| qPDB1-R | ACAATTTCAGCACCAACACCAAATGATG |
| qLAT1-F | CTGTTGCCACACCAACAGGATTATTGA |
| qLAT1-R | ACCTTGGAATTCCTCTGGTGCCAATT |
| qPKP1-F | CAGACGATGACGAGCTGTACTTACGGAT |
| qPKP1-R | GCCGGGCAGATCTGTAGACTCAGAGT |
| qPKP2-F | TTCCCATGCCAGAATTTATTATCGAAGG |
| qPKP2-R | TCCCTTACGAATATAGTGTTTCATGGTTGC |
